# Nerve Growth Factor Secretion From Pulp Fibroblasts is Modulated by Complement C5a Receptor and Implied in Neurite Outgrowth

**DOI:** 10.1038/srep31799

**Published:** 2016-08-19

**Authors:** Fanny Chmilewsky, Warda Ayaz, James Appiah, Imad About, Seung-Hyuk Chung

**Affiliations:** 1 Department of Oral Biology, University of Illinois at Chicago, Chicago, Illinois 60612, USA; 2 Aix-Marseille Université, CNRS, ISM UMR 7287, 13288, Marseille cedex 09, France

## Abstract

Given the importance of sensory innervation in tooth vitality, the identification of signals that control nerve regeneration and the cellular events they induce is essential. Previous studies demonstrated that the complement system, a major component of innate immunity and inflammation, is activated at the injured site of human carious teeth and plays an important role in dental-pulp regeneration via interaction of the active Complement C5a fragment with pulp progenitor cells. In this study, we further determined the role of the active fragment complement C5a receptor (C5aR) in dental nerve regeneration in regards to local secretion of nerve growth factor (NGF) upon carious injury. Using ELISA and AXIS co-culture systems, we demonstrate that C5aR is critically implicated in the modulation of NGF secretion by LTA-stimulated pulp fibroblasts. The NGF secretion by LTA-stimulated pulp fibroblasts, which is negatively regulated by C5aR activation, has a role in the control of the neurite outgrowth length in our axon regeneration analysis. Our data provide a scientific step forward that can guide development of future therapeutic tools for innovative and incipient interventions targeting the dentin-pulp regeneration process by linking the neurite outgrowth to human pulp fibroblast through complement system activation.

Dental caries is one of the most common human pathologies, affecting 90% of the U.S. adult population^1^. The complexity of the dentin-pulp complex response to carious injury is directly correlated to its severity^2^. A protective reactionary dentin is produced beneath the injured site by surviving odontoblasts in a moderate carious injury^3^. Deeper injuries require more elaborated biological processes, which imply the regeneration of a full part of dentin-pulp complex including the vascularization, innervation and the production of a reparative dentin by a new generation of odontoblast-like cells^4^. In both cases, nerve fiber sprouting is observed in the surviving pulp beneath the injured site^5^, suggesting that the sprouting process is required for efficient dentin-pulp regeneration. These regenerated nerves can enhance regeneration and healing by induction of immune cell recruitment and neoangiogenesis at the injured site^6^,^7^. Proper nerve regeneration is also extremely important for maintaining pulp integrity and sensory function^8^. However, little is known about the initial mechanisms that regulate dental pulp nerve sprouting and the subsequent regenerative process. The duration and extension of dental nerve regeneration are regulated by the spatio-temporal modulation of neurotrophin expression at the cellular level^5^,^9^. Several studies conducted in the rat model suggest that nerve growth factor (NGF) expression in pulp fibroblasts is up-regulated after tooth injury^10^,^11^.

The complement system, which is one of the first responses of innate immunity, has been recently identified as an important mediator of tissue regeneration. This function seems especially supported by the active fragment C5a, which exerts its action through the interaction with the C5a receptor (C5aR), as demonstrated in the liver, bone and cardiac tissues^12^,^13^,^14^. Similarly, there is compelling evidence that complement system activation is involved in the early steps of dentin-pulp regeneration. Indeed, Chmilewsky *et al*. demonstrated for the first time that the complement system is activated at the injured site of human carious teeth and may play an important role in dental-pulp regeneration^15^,^16^,^17^,^18^. The complement active fragment C5a, which is known to be activated from plasma proteins, induces the recruitment of human pulp progenitor cells to the injured site. This represents one of the initial steps of dentin-pulp regeneration^19^,^20^. Pulp fibroblasts have previously been identified as source of regeneration signals in case of traumatic injury^21^. However, their capacity to secrete and guide nerve growth signals in response to caries has not been studied extensively. To date, no investigation has systematically explored the effect of the complement system on pulp fibroblasts and the subsequent effect of this interaction on dental nerve regeneration.

We recently identified the role of C5aR in carious tooth nerve regeneration. The pulp fibroblasts controlled the brain-derived neurotrophic factor (BDNF)-mediated nerve regeneration through C5aR activation upon carious injury^18^. In this study, we further investigated the role of the C5aR in dental nerve regeneration in regards to local production of NGF upon carious injury. Our data show that C5aR is critically implicated in the NGF secretion modulation by LTA-stimulated pulp fibroblasts and this modulation regulates neurite outgrowth length in our co-culture nerve regeneration device.

## Results

### C5aR expression is increased in LTA-stimulated human pulp fibroblasts and under carious injury

**Figure 1:**
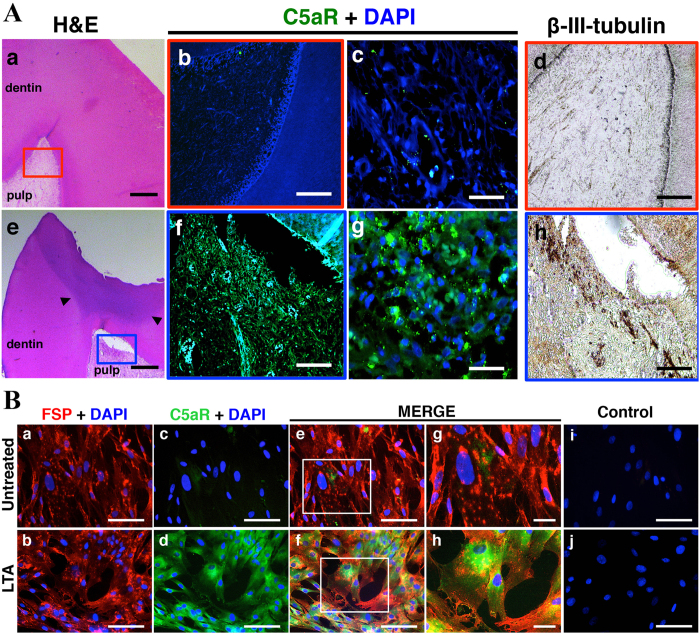
Expression of C5aR by human pulp cells *in vivo* and *in vitro*. (**A**) C5aR and nerve densification are co-localized in carious tooth sections. C5aR and beta-III tubulin are detected by immunohistochemistry in intact (a) and carious teeth (e). While C5aR is not detected in intact teeth (b,c), its labeling is intense in carious teeth beneath the injury (f,g). A uniform and scattered distribution of the ß-III-tubulin is detected in intact teeth (d), a densification of this staining is observed beneath carious injured sites (h). Nuclei were counter-stained with DAPI (blue) (b,c,f,g). Symbols: arrowheads indicate caries lesion. Scale bars: a,e = 500 μm; b,d,f,h = 200 μm; c,g = 50 μm. (**B**) C5aR is expressed by LTA-stimulated human pulp fibroblasts *in vitro*. Immunofluorescence double staining was used to analyze C5aR expression in human pulp cells. Fibroblast surface protein (FSP) is detected on the surface of all human pulp cells (a,b). While few pulp fibroblasts express C5aR in untreated condition (c,e,g), an intense C5aR staining is detected after LTA-stimulation (d,f,h). For each condition, negative controls, performed by replacement of the FSP and C5aR primary antibodies with isotype controls, showed no labeling (i,j). Secondary antibody used to detect FSP was Alexa-594 (red), C5aR detection was done with Alexa-488 (green); Nuclei were counter-stained with DAPI (blue). g,h magnified respectively from the box in e,f. Scale bars: a–f,i,j = 50 μm; g,h = 25 μm.

To identify the role of C5a in dental nerve regeneration, we first investigated the expression of C5aR both in intact and carious tooth sections. In intact tooth sections (Fig. 1Aa), very little or none of C5aR was observed in pulp tissue by anti-C5aR immunohistochemistry (Fig. 1Ab,c). However, strong C5aR immunoreactivity was detected throughout the carious tooth sections beneath the injury area (Fig. 1Ae–g). Nerve fibers were localized in the same tooth sections using ß-III-tubulin antibody, a marker of central and peripheral nerve fibers^22^. Uniform and scattered ß-III-tubulin expression was detected throughout the intact section (Fig. 1Ad). In carious tooth sections, strong C5aR immunoreactivity was detected throughout the carious tooth sections beneath the injury area (Fig. 1Ae–g). Nerve fiber component staining was prominent and intense right beneath the injury area (Fig. 1Ah) indicating that nerve fiber sprouting is present in carious pulp tissues. These results show a spatial correlation between the expression of C5aR and nerve sprouting in human carious teeth.

We next utilized lipoteichoic acid (LTA), which is a major component of the cell wall of Gram-positive bacteria and inflammation inducer to mimic dental caries *in vitro*^23^. Human pulp cell double stained using anti-FSP, as a fibroblast marker^24^ and anti-C5aR show that very few pulp fibroblasts expressed C5aR (Fig. 1Ba–g). However, all pulp fibroblasts did express C5aR after LTA treatment (Fig. 1Bb–h). These results, which specifically identify pulp fibroblasts as cells that are able to express C5aR under carious injury, are consistent with our *in vivo* data (Fig. 1A).

### NGF is expressed in human pulp fibroblasts and C5aR expression is increased under LTA-stimulation

**Figure 2:**
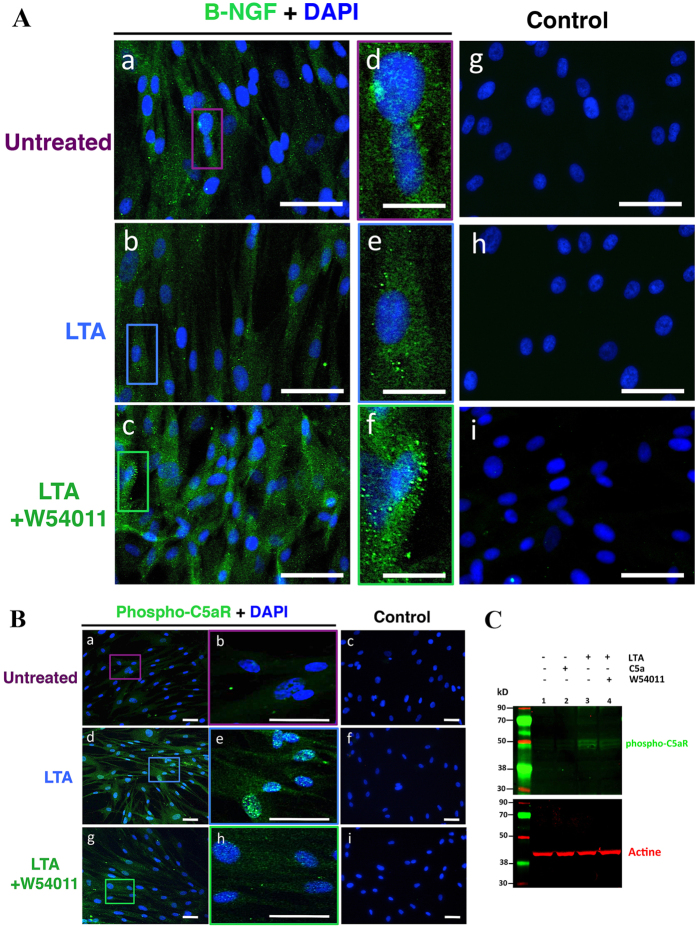
Detection of ß -NGF and phosphor-C5aR in human pulp fibroblasts. (**A**) Representative images of ß-NGF immunostaining in human pulp fibroblasts incubated with or without LTA and with or without C5aR specific antagonist (W54011) for 48 hours. The ß-NGF detected in untreated pulp fibroblasts (a,d) seems to be decreased by stimulation with LTA (b,e). Co-incubation of LTA-stimulated pulp fibroblasts with W54011 increased the ß-NGF staining, and the fluorescence detected seems also to be higher than in untreated conditions (c,f). No ß-NGF staining was observed in the isotype controls (g–i). Secondary antibody used to detect ß-NGF was Alexa-488 (green); Nuclei were counter-stained with DAPI (blue). d,e,f magnified respectively from purple, blue and green boxes in a, b and c. Scale bars: a–c,g–i = 50 μm d–f = 15 μm. (**B**) Representative images of phospho-C5aR immunostaining in human pulp fibroblasts incubated with or without LTA and with or without C5aR specific antagonist (W54011) for 48 hours. An intense phospho-C5aR staining is observed in LTA-stimulated pulp fibroblasts (d,e) compared to untreated conditions (a,b). The phosphor-C5aR staining detected in LTA-stimulated pulp fibroblasts was reduced by their co-incubation with W54011 (g,h). No phospho-C5aR staining was observed in the isotype controls (c,f,i). b,e,h magnified respectively from box in a,d,g. Scale bars: a,c,d,f,g,i = 50 μm d,e,h = 15 μm. (**C**) Western blot analysis of phospho-C5aR in human pulp fibroblasts. Total lysates from pulp fibroblasts incubated with or without LTA, C5a and W54011 were analyzed by Western blot using antibodies against C5aR(pSer338). β-actin was immunoblotted to ensure similar gel loading of the starting material in each sample. Very low levels of C5aR(pSer338) were detected in unstimulated (column 1) and C5a-stimulated pulp fibroblasts (column 2). However, the quantity of C5aR(pSer338) detected in pulp fibroblasts was increased by their stimulation with LTA (column 3). This level of C5aR phosphorylated was decreased by the co-incubation of LTA-stimulated pulp fibroblasts with W54011 (column 4).

The NGF has been suggested as a major nerve growth signal and several studies reported its up-regulation in pulp tissues after tooth trauma and injury^10^,^11^. Thus we next investigated NGF expression in intact and LTA-stimulated human pulp fibroblasts, through ß-NGF immunofluorescent staining. The NGF expression was observed in untreated pulp fibroblasts (Fig. 2Aa,d). Immunoreaction product is deposited with a uniform punctate pattern localized in the cytoplasm of cells. It seems that the expression level is decreased after 48 hours of LTA treatment (Fig. 2Ab,e). Interestingly, the co-incubation of LTA-stimulated pulp fibroblasts with W54011, the C5aR specific antagonist, increased the ß-NGF staining (Fig. 2Ac,f), as compared to both untreated (Fig. 2Aa,d) and LTA-treatment (Fig. 2Ab,e) groups.

The C5aR activation has been visualized through the detection of C5aR phosphorylated form (Fig. 2B). While no specific staining was detected in unstimulated-pulp fibroblasts (Fig. 2Ba,b), an intense phospho-C5aR staining is detected in pulp fibroblasts after 48 hours of LTA-stimulation (Fig. 2Bd,e). This activation is reduced by co-incubation of cells with both LTA and W54011 (Fig. 2Bg,h). Western blot analysis confirms that a very low level of C5aR was phosphorylated in unstimulated pulp fibroblasts (Fig. 2C, column 1). The addition of recombinant C5a didn’t affect the level of C5aR phosphorylated in pulp fibroblasts, confirming that very few C5aR are expressed in unstimulated pulp fibroblasts (Fig. 2C, column 2). However, high level of C5aR phosphorylated was detected 48 hours after LTA stimulation (Fig. 2C, column 3), indicating that C5aR is expressed and activated in LTA-stimulated pulp fibroblasts. As expected, the phosphorylation of C5aR in LTA-stimulated fibroblasts was reduced by their co-incubation with C5aR specific antagonist (Fig. 2C, column 4).

Taken together, these data indicate that there is a negative correlation between the secretion of NGF by human pulp fibroblasts and C5aR activation.

### C5aR acts as a negative regulator of the β-NGF secretion by LTA-stimulated fibroblasts

**Figure 3:**
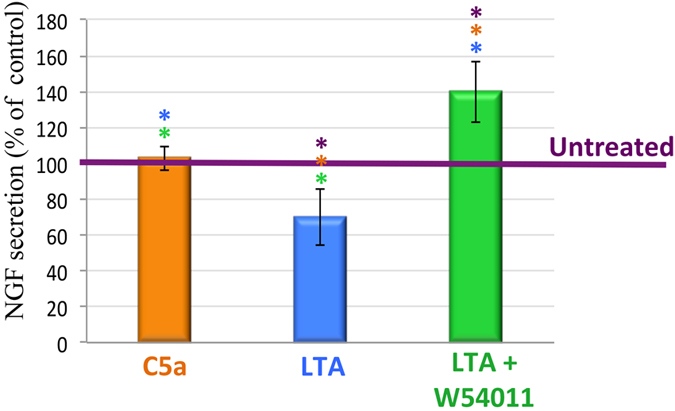
ß-NGF secretion by human pulp fibroblasts. The quantities of ß-NGF detected in the supernatant of untreated pulp fibroblasts after 48 hours was arbitrarily fixed at 100% reference point for each experiment, and is indicated by a purple line in the graphic (**untreated**). As expected, the co-incubation of untreated pulp fibroblasts with recombinant C5a (**C5a**) has no effect on the ß-NGF secretion (102.86 ± 6.71%). However, LTA stimulation of pulp fibroblast for 48 hours (**LTA**) leads to a significant decrease of ß-NGF in the supernatant (70.01 ± 15.65%). The use of W54011 (**LTA + W54011**), a C5aR specific antagonist, significantly increased the quantity of ß-NGF detected in the supernatant of LTA-stimulated pulp fibroblasts (139.79 ± 16.85%). *P < 0.05. n = 3 per group.

The quantification of ß-NGF in the supernatant of pulp fibroblast was measured by enzyme-linked immunosorbent assay (ELISA) after 48 hours of treatment with recombinant C5a, LTA and LTA combined with W54011 (Fig. 3). In concordance with our previous data showing that very few pulp fibroblasts express the C5aR, the stimulation of these cells with recombinant C5a did not affect the basal level of β-NGF secretion (orange histogram versus purple line, the purple line indicates an untreated condition fixed at 100%). However, the stimulation with LTA for 48 hours leads to a significant decrease of the β-NGF***-***quantity detected in cell supernatants (blue histogram versus purple line). Interestingly, the co-incubation of LTA-stimulated fibroblasts with the C5aR specific antagonist significantly increased the secretion of β-NGF at 48 hours (green histogram versus blue histogram and purple line). These results are consistent with our NGF immunocytochemistry data shown in Fig. 2 and demonstrate that C5aR acts as a negative regulator of β-NGF secretion in LTA-stimulated fibroblasts.

### NGF secretion is controlled by C5aR under LTA-stimulation and is implicated in neurite outgrowth

As the C5a/C5aR interaction in LTA-stimulated pulp fibroblasts appears to be implicated in the modulation of NGF secretion, we next investigated the direct effect of this interaction with the NGF modulation in neurite outgrowth process. The neurite outgrowth experiments were performed with the Axon Investigation Systems (AXIS). The AXIS device has been successfully used in several recent studies and proved its accuracy and efficacy for nerve outgrowth assay^25^,^26^.

**Figure 4:**
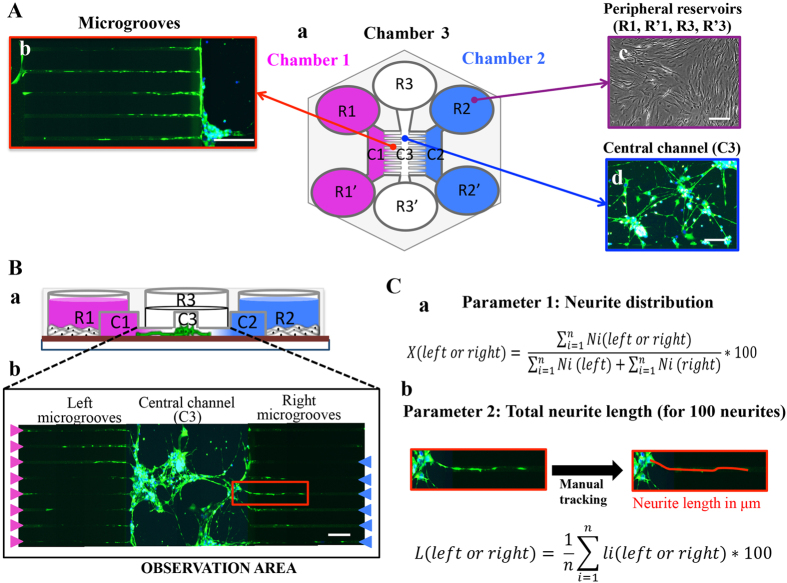
Neurite outgrowth experiment assay and analysis. (**A**) a: The neurite outgrowth device is made of two peripheral chambers (left and right chambers) and a central chamber. Each of these chambers is composed by two reservoirs (R1 and R1′; R2 and R2′; R3 and R3′) interconnected by a thin channel (respectively C1, C2 and C3). C3 communicates with C1 and C2 through thin microgrooves (500 × 10 × 5 μm). Human neurons were seeded into C3 (d); human pulp fibroblasts were seeded into the reservoirs of the 2 peripheral chambers, i.e. R1, R1′, R2 and R2′ (c). C1 and C2 were left empty of cells. Neurons extend neurites toward C1 and C2 through migrogrooves (b). Scale bars: b = 100 μm; c = 200 μm; d = 50 μm. (**B**) (a) An AXIS cross-section showing that devices are disposed on glass slide previously coated with poly-D-lysine. Pulp fibroblasts of the right chamber were incubated ±LTA, ±C5a and ±W54011 in serum-free medium. Human neurons of the central chamber were incubated in neural medium ± AG-879 (a TrkA antagonist). (b) Fluorescent microscopic view of an observation area: Neurite outgrowth assays were run for 48 hours and a β-tubulin immunostaining, a minimum of 8 observation areas by experiment was reconstituted from 6 successive pictures taken on the same x-axis. Pink arrowheads indicate the presence of 1 neurite inside a microgroove on the left side; Blue arrowheads indicate the presence of neurite inside a microgroove on the left side. Scale bar = 100 μm. (**C**) Neurite outgrowth analysis: (a) Analysis of the neurite distribution: (b) Analysis of the total neurite length. For more details, please see ‘METHODS’ section.

This device consists of distinct chambers and these chambers are designed to cultivate different cells separately (Fig. 4). Each chamber is composed of two wells interconnected by a thin channel. The manufacturer’s instructions are slightly modified and adapted to our study. Devices were assembled on glass slide coated with poly-D-Lysine according the manufacturer’s instructions. Human neurons were seeded into the central chamber (2.0 × 10^4^ cells) in neuronal medium.

**Figure 5:**
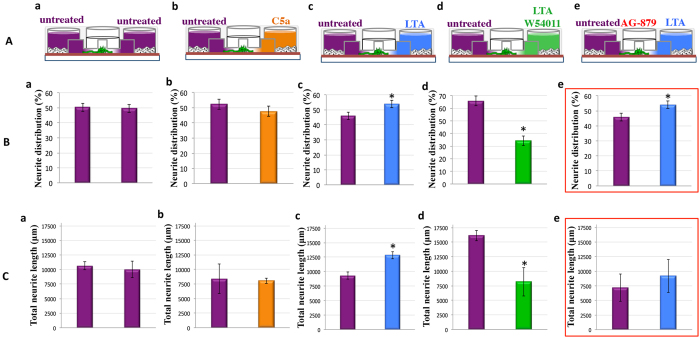
Implication of C5aR and ß-NGF in neurite outgrowth. (**A**)—Schematic representations of neurite outgrowth assays (a–e). (**B**)—Statistical analysis of neurite distribution. Normally neurites are equally distributed toward left and right chamber (**untreated**: 50.33 ± 2.66% vs. **untreated**: 49.67 ± 2.66%) (a). This result is not affected when pulp fibroblasts of the right reservoir are co-incubated with recombinant C5a (**untreated**: 52.3 ± 3.30% vs. **C5a**: 47.7 ± 3.30%) (b). When pulp fibroblasts of the right chamber were stimulated with LTA, the number of neurites toward the stimulation is significantly higher than untreated fibroblasts (**untreated**: 46.0 ± 2.52% vs. **LTA**: 54.0 ± 2.52%) (c). The co-incubation of LTA-stimulated pulp fibroblasts with W54011 drastically reduced the number of neurite detected in the direction of the stimulation (**untreated**: 65.8 ± 3.77% vs. **LTA + W54011**: 34.2 ± 3.77%) (d). The incubation of neurons with AG-879, a Trka specific antagonist, has no effect on the number of neurites detected toward LTA stimulation (**untreated**: 45.89 ± 2.63% vs. **LTA**: 54.11 ± 2.63%) (e). (**C**)–Statistical analysis of neurite lengths:. No difference between the neurite length toward the right and the left reservoirs was observed (**untreated**: 10657.97 ± 696.69 μm vs. **untreated**: 10022.33 ± 1454.31 μm) (a). This result is not affected when pulp fibroblasts of the right reservoir are co-incubated with recombinant C5a (**untreated**: 8411.13 ± 2564.23 μm vs. **C5a**: 8067.37 ± 457.39 μm) (b). When pulp fibroblasts of the right chamber were stimulated with LTA, the total neurite length in direction of the stimulation is significantly higher than the total neurite length obtained in direction of untreated fibroblasts (**untreated**: 9309.43 ± 599.47 μm vs. **LTA**: 12813.33 ± 599.34 μm) (c). The co-incubation of LTA-stimulated pulp fibroblasts with W54011 drastically reduced the length of neurite towards the stimulation (**untreated**: 16135.67 ± 895.92 μm vs. **LTA + W54011**: 8199.94 ± 2457.50 μm) (d). The neurite length increase in direction of LTA stimulation (c) was abolished by the incubation of neurons with AG-879 (**untreated**: 7202.61 ± 2349.44 μm vs. **LTA**: 9196.83 ± 2818.06 μm) (e).

When pulp fibroblasts were seeded in the AXIS’s peripheral chambers (Fig. 5Aa), the same number of neurites was detected inside microgrooves over 48 hour period toward the two sides (Fig. 5Ba), and the total neurite lengths toward both sides were equal (Fig. 5Ca). These data were not affected when the pulp fibroblasts of one of the lateral chamber were incubated with recombinant C5a (Fig. Ab,Bb,Cb). However, when pulp fibroblasts of one of the lateral chamber were stimulated with LTA (Fig. 5Ac), both the number of neurites (Fig. 5Bc) and the total neurite lengths (Fig. 5Cc) were significantly higher toward LTA stimulation. Interestingly, the co-incubation of LTA-stimulated pulp fibroblasts with W54011 abolished this neurite outgrowth (Fig. 5Ad,Bd,Cd). These results demonstrate that the increase of neurite outgrowth in direction of LTA-stimulated pulp fibroblasts is dependent of the activation of C5aR.

NGF is known as neurite outgrowth inducers through the tropomyosin receptor kinase A (TrkA)^27^,^28^. Human neurons were incubated with TrkA specific antagonist AG-879 to examine the direct effect of NGF on neurite outgrowth (Fig. 5Ae). Interestingly, while the use of AG-879 did not affect the number of neurite detected in the direction of LTA stimulation (Fig. 5Be as compared to Fig. 5Bc: significant difference between purple and blue bars is conserved), it significantly decreased their length (Fig. 5Ce as compared to Fig. 5Cc: significant difference between purple and blue bars observed in 5Cc is abolished in 5Ce). These results indicate that the secretion of NGF by pulp fibroblasts under LTA-stimulation, although negatively regulated by C5aR activation, is involved in the increase of neurite extension length in the direction of LTA-stimulation chamber.

## Discussion

Our study demonstrates that human pulp fibroblasts localized beneath carious injuries *in vivo* and under LTA-stimulation *in vitro* express the C5aR. Further, we identify this newly expressed C5aR as a negative regulator of NGF secretion by LTA-stimulated pulp fibroblasts. Finally, we found that the NGF secreted by LTA-stimulated pulp fibroblasts, although negatively regulated by C5aR activation, is involved in the control of the neurite outgrowth length in the direction of injury. Together with this study, we recently reported the C5aR-mediated nerve regeneration through BDNF secretion modulation^18^. We showed that C5aR functions as a positive regulator for BDNF secretion and this modulation has a direct role in regenerative nerve outgrowth toward to the injury site^18^. Thus our two studies may reveal the first glimpse of the mechanisms that C5a could be one of the initial signals in dental nerve sprouting upon carious injury and further controls nerve regeneration by inducing the local production of neurotrophins.

It has long been established that tissue regeneration is closely linked to inflammation. For example, the application of corticosteroids in myocardial infarction significantly reduced the inflammation and inhibited the tissue regeneration^29^. Interestingly, C5a, which is a crucial component of innate immunity, has been recently identified as an important mediator of tissue regeneration^12^,^13^,^14^. Interestingly, our immunohistochemistry results revealed an intense C5a receptor staining in the inflamed pulp localized just beneath the injury of carious teeth. The C5a, through its interaction with the C5aR, is mainly known to be a powerful chemotactic factor for immune cells, such as neutrophils, eosinophils, basophils/mastocytes, monocytes/macrophages, dendritic cells, and B and T lymphocytes^30^,^31^. The implication of C5a in regenerative process has been supported by the report of C5aR expression, either constitutively or in response to a stimuli, by various non-immune cells such as endothelial cells, astrocytes, cells from skin, intestine, and heart, and human mesenchymal stem cells^32^. Consistent with these data, we found that pulp fibroblasts express the C5aR under LTA-stimulation. Several studies support the idea that C5a is able to modulate the molecular composition of the environment by controlling the liberation of various molecules by cell expressing the C5aR. For example, it has been established that C5a controls the histamine and TNFα liberation by mast cells and basophils, the vascular endothelial growth factor secretion by Retinal muller cells^33^, or the NGF over-expression by both human glioblastoma cell line and rat astrocytes^34^. Interestingly, it became evident that dentin-pulp regeneration is a process orchestrated by the local control of growth factor expression at pulp cell level^4^,^35^. Surprisingly, the ability of C5a to modulate the liberation of dentin-pulp regeneration signals has not received much attention. So far, only one study conducted in our laboratory, demonstrated that under carious injury the C5aR constitutes a positive regulator for the BDNF neurotrophin secretion and that this modulation has a direct role in regenerative nerve outgrowth toward the injury site^18^. Since their initial discovery, neurotrophins are of central importance both in normal CNS and PNS development and neuronal regeneration capacity for axon outgrowth in neurological diseases^36^. It is now generally accepted that up-regulation of several neurotrophins after injury involves a critical step in nerve regeneration^37^. Previous studies conducted in the rat model suggest that NGF expression in pulp fibroblasts is up-regulated shortly and transitorily after tooth injury and this up-regulation might be responsible for dental nerve sprouting^10^,^11^,^38^. In this study we clearly demonstrated, using both immunofluorescent staining on human pulp fibroblasts and ELISA on their supernatant, that the secretion of NGF by these fibroblasts is significantly reduced 48 h after LTA-stimulation. Although apparently in opposition with the previous publications^8^,^38^, our results do not excluded the possibility of a transitory increase of NGF secretion by pulp fibroblasts during the first 48 hour. Interestingly, the use of a C5aR specific antagonist (W54011), which drastically reduced the presence of C5aR-active form in LTA-stimulated fibroblasts as revealed our phospho-C5aR immunostaining and western blot analysis, significantly increased the secretion of NGF by LTA-stimulated pulp fibroblasts. These results indicate that C5aR constitute a negative regulator of the NGF secretion by LTA-stimulated pulp fibroblasts. It is surprising that C5aR regulates two major neurotrophins – BDNF^18^ and NGF by pulp fibroblasts under LTA stimulation in opposite ways. The NGF was initially reported for its role in extensive axonal growth in the CNS^39^. Consistent with these data, the use of AG879, a TrkA specific antagonist in our neurite outgrowth experiments clearly showed that the NGF secreted by LTA-stimulated pulp fibroblast, although negatively regulated by C5aR activation, is involved in the neurite outgrowth toward the LTA-stimulation. In this study we decomposed the neurite outgrowth in 2 distinct parameters: the number of neurite recruited and the length of the neurite extension. Surprisingly, while the use of AG879 did not affect the number of neurite detected in the direction of LTA stimulation but only significantly decreased their length, the co-incubation of LTA-stimulated pulp fibroblasts with a specific C5aR antagonist drastically reduced these 2 parameters. Thus, our results suggest that activating the C5aR complement pathway is more beneficial in dental nerve regeneration under carious injury. The importance of C5aR activation in dental nerve regeneration was suggested by our *in vivo* results, demonstrating a spatial correlation between the nerve sprouting and C5aR in human carious teeth beneath the injured site. The activation of C5aR signaling results in BDNF secretion by pulp fibroblasts that induces prominent neurite outgrowth toward the site of carious injury^18^. Our current NGF data supports that inhibition of C5aR can induce an increased NGF secretion and has a role in neurite length extension. This discrepancy might be due to that NGF has more significant roles in neurogenesis and the survival of existing neuronal cells though it also has a moderate effect in nerve outgrowth in regenerative pulp^40^,^41^.

Taken together, we provide data demonstrating that human pulp fibroblasts express C5aR in association with carious injury both *in vivo* and after LTA-stimulation *in vitro*. We show that caries-associated C5aR expression is followed by C5a activation, with C5a generated from pulp fibroblasts following complement activation. C5aR signaling results in NGF secretion by pulp fibroblasts that induces prominent neurite outgrowth toward the site of carious injury. These steps represent the body’s initial responses to pulp caries and the initiation of protective/reparative dentinogenesis. Our study provide new data to the initial step of dentin-pulp regeneration process and the poorly understood regulation of nerve fiber sprouting observed in response to tooth injuries. In this regard, our project could bring significant new knowledge essential for elaborating future dentin-pulp engineering strategies.

## Methods

### Materials

Cell culture materials and reagents were from Corning (Manassas, VA, USA). Recombinant C5a and TrkA specific antagonist (AG-879) were from R&D Systems (Minneapolis, MN, USA). Anti-ß III tubulin and anti-Fibroblast Surface Protein (FSP) were from Abcam (Cambridge, UK). Anti-C5aR and anti-β-actin antibodies were respectively from Proteintech (Chicago, IL, USA) and BioLegend (San Diego, CA, USA). Fluorescent secondary antibodies were from Life Technologies (Grand Island, NY, USA), HRP-conjugated secondary antibody was from KPL (Gaithersburg, MD, USA) and IRDye secondary antibodies were from LI-COR (Lincoln, NE, USA). Low-fluorescence PVDF transfer membranes were from Thermo Scientific (Waltham, MA, USA). The C5aR antagonist (W54011), and chambers to evaluate the neurite outgrowth (AXIS) were from EMD Millipore (Darmstadt, Germany). The human beta NGF ELISA kit and lipoteichoic acid (LTA) were from Sigma-Aldrich (St. Louis, MO, USA), and chemicals were from Fisher Chemical (Nazareth, PA, USA). Human neurons, neuronal medium and neuronal growth supplement were from Sciencell (Carlsbad, CA, USA). All experimental protocols used for this study were in accordance with the guidelines according to the Institutional Animal Care and Use Policy and approved by the IRB Protocol Committee at the University of Illinois at Chicago.

### Molar Teeth Collection

Human immature third molars, freshly extracted for orthodontics reasons, and carious teeth were obtained in compliance with French legislation (informed patients’ consent and institutional review board approval of the protocol at the Aix-Marseille Université used).

### Immunohistochemistry

Teeth were fixed and routinely processed as previously described^20^. Seven-micrometer-thick paraffin tissue sections from intact and carious teeth were deparaffinised with xylene and graded ethanol. Some sections were hematoxylin-eosin-stained. For other sections, an antigen retrieval was performed at 98 °C for 20 minutes in Tris 1 mM/0.1 mM EDTA/0.5% Tween, pH 6.0 and nonspecific binding sites were blocked with 0.25% caseine in PBS for 15 minutes at room temperature. Then tooth sections were incubated for 1 hour at room temperature with mouse anti-human C5aR (20 μg/mL) or rabbit anti-beta III tubulin (1 μg/mL) or their respective isotype control.

The C5aR immunostaining was done by incubating section for 45 minutes with Alexa Fluor-488 rabbit anti-mouse IgG (2 μg/mL), and DAPI (1 μg/mL) counterstain for fluorescence microscopy.

Simultaneously, beta III tubulin immunostaining was done by incubating tooth sections for 2 hours at room temperature with HRP-conjugated goat anti-rabbit (1:200). Diaminobenzidine (DAB, 0.5 mg/mL) was used to visualize the reaction.

### Cell Cultures

Human pulp cells were prepared from immature third molars at the 2/3 root formation stage by the explant outgrowth method^42^. The teeth were obtained from three different donors for each experiment (4 molars/donor). Human pulp cells were cultured at 37 °C under 5% CO_2_ in Dulbecco’s Modification of Eagle’s Medium (DMEM) with 4.5 g/L glucose, L-glutamine, sodium, pyruvate supplemented with 10% heat-inactivated fetal bovine serum, 100 μg/mL streptomycin, 100 U/mL penicillin.

Primary human neuronal cells were cultured at 37 °C under 5% CO_2_ in Neuronal Medium, which consist of Neuronal basal medium supplemented with neuronal growth supplement (NGS), 100 μg/mL streptomycin, and 100 U/mL penicillin.

### Immunofluorescence Staining

Human pulp cells were seeded overnight at 10 × 10^3^cells/well on eight-well glass culture chambers before being stimulated with or without of LTA (10 μg/mL) for 24 hours. After being washed, cells were fixed with 4% paraformaldehyde during 10 minutes and permeabilized with 0.3% Tween-20/0.3% glycine in PBS for 30 minutes. Then, nonspecific binding sites were blocked with 3% BSA for 1 hour. Cells were incubated simultaneously with rabbit anti-human C5aR (10 μg/mL), anti-phospho-C5aR(pSer338) (10 μg/mL) and mouse anti-human FSP (20 μg/mL) or their respective control isotypes for 1 hour. After washing, the cells were incubated for 40 minutes with a mix of Alexa Fluor-594 donkey anti-mouse IgG, Alexa Fluor-488 goat anti-rabbit IgG (2 μg/mL), and DAPI (1 μg/mL). After washing the coverslips were sealed and photographs taken using a Leica DMI6000 B fluorescent microscope.

### Neurotrophin quantification

Human pulp cells were grown in 60 mm Petri dish. At subconfluency cells were incubated in 1.5 mL of medium containing or not 10 μg/ml of LTA. Simultaneously, cells were incubated with or without 10 nmol/L of W54011, a C5aR specific antagonist, and with or without 200 ng/mL of recombinant C5a After 48 hours pulp cell supernatants were harvested and β-NGF concentration was immediately determined by enzyme-linked immunosorbent assay (ELISA) according to the manufacturer’s instructions.

### Western blotting

Human dental pulp fibroblasts were stimulated with or without of LTA (10 μg/mL) for 48 hours. Cells were trypsinized and harvested by centrifugation at 1,500 rpm for 5 minutes. The protein lysate samples will be then prepared from the cell pellets that will be solubilized in lysis buffer (150 mM NaCl, 50 mM Tris pH8, 1% Triton, 0.1% SDS). After incubation on ice for 30 minutes, the cell lysate samples were centrifuged at 12,000 rpm at 4 °C for 15 min to collect the supernatant. Fifty micrograms of total protein were separated on 12% polyacrylamide gel and then transferred onto Low-Fluorescence PVDF transfer membrane. The membranes were blocked with 5% BSA for 2 hours and blotted with anti-human C5aR(pSer338) or anti-β-actin antibody (1 μg/ml). Then blots were incubated 1 hour either with IRDye-800CW (green) or with IRDye-680LT (red) secondary antibodies following the manufacture’s instruction. Finally, the blots were washed and immunodetected proteins were visualized and analysed with the optical scanner Odyssey CLx (Lincoln, NE, USA).

### Neurite outgrowth assay

Neurite outgrowth assay was investigated using AXon Investigation Systems (AXIS) (Fig. 4). This device consists of three distinct chambers (1 central and 2 peripheral chambers), each composed of two wells interconnected by a thin channel. Channels are separated from each other by microgrooves preventing cell bodies from flowing through while allowing neurite passage. The manufacturer’s instructions were slightly modified and adapted to our study. Devices were assembled on glass slide (25 × 75 mm) coated with poly-D-Lysine (0.5 mg/mL) according the manufacturer’s instructions. Human Neuronal Cells were seeded within the channel of the central chamber (2.0 × 10^4^ cells) in Neuronal Medium containing 5 μmol/mL of TrkA specific antagonist (AG-879). Wells of the 2 peripheral chambers were filled with human pulp cells in DMEM (2.0 × 10^5^ cells)^±^ LTA (10 μg/ml). Simultaneously, human pulp cells were incubated either with or without W54011 (10 nmol/L). After 48 hours at 37 °C and 5% CO2, cells were fixed with 4% paraformaldehyde during 10 minutes. Then cells were permeabilized with 0.3% Tween-20/0.3% glycine in PBS for 30 minutes and saturated for 1 hour with 3% BSA in PBS. Cells were incubated overnight at 4 °C with rabbit anti-beta II tubulin (1 μg/mL), or their respective isotypes. Finally, cells were incubated for 45 minutes with Alexa Fluor-488 mouse anti-rabbit IgG (2 μg/mL) and DAPI (1 μg/mL). Photographs were took using a Leica DMI6000 B fluorescent microscope, and neurite outgrowth inside microgrooves from the central chamber toward peripheral chambers was analyzed using ImageJ 1.43 software (NIH, Bethesda, MD).

### Neurite outgrowth analysis

The neurite outgrowth analysis was performed by the manual tracking of all neurites detected inside microgrooves of both sides, and start from neuron nucleus to terminal neurite extension. Then, neurite trajectories are reported at the point (0; 0) of a graphic representing the length in micrometer. When the neurite outgrowth is directed toward the right chamber, the length is arbitrarily considered as positive value and as negative value when neurite outgrowth is directed toward the left chamber. So, for each experiment the average of all neurite lengths provide a dot corresponding to the global direction of the neurite outgrowth; a dot shifted to the graphic right indicate that the neurite outgrowth is mainly directed toward the right chamber. The neurite distribution is expressed in percentage and represents the percent of neurites detected toward the left and the right sides. X(left or right) (Fig. 4Ca): percent of neurites detected toward the left or toward the right; n: number of observation area by experiments, n ≥ 8; Ni(left or right): number of neurites detected toward the left or toward the right in each observation area (represented by pink and blue arrowhead in Fig. 4B). Analysis of the total neurite length: For each experiment, the total neurite length toward the right as well as toward the left is expressed for a total of 100 neurites. The length of each neurite detected inside microgroove is given by a manual tracking. Manual tracking of neurite growth was performed from the neuron nucleus to terminal neurite extension, which is localized either inside microgroove or inside C1/C2. L(left or Right): total length of neurite detected toward the left side or toward the right side; n: number of observation area by experiments, n ≥ 8; l(left or right): sum of neurite length detected toward the left or toward the right in one observation area (Fig. 4Cb).

### Statistical analysis

All experiments were repeated at least 3 times, and statistical significance was determined using the Student’s *t* test to compare the different treatments and their respective controls. Data were expressed as means ± SD and considered significant for *P* < 0.05.

## Additional Information

**How to cite this article**: Chmilewsky, F. *et al*. Nerve Growth Factor Secretion From Pulp Fibroblasts is Modulated by Complement C5a Receptor and Implied in Neurite Outgrowth. *Sci. Rep.* **6**, 31799; doi: 10.1038/srep31799 (2016).
